# Cell-Based Therapies for Post-Traumatic Ankle Osteoarthritis and Osteochondral Lesions of the Talus: A Systematic Scoping Review of an Emerging and Heterogeneous Evidence Base

**DOI:** 10.3390/bioengineering13070843

**Published:** 2026-07-22

**Authors:** Se Yeong Jeon, Min Woo Kim, Dong Ha Lee

**Affiliations:** 1 Science and Technology Department, Ulsan National Institute of Science and Technology, Ulsan 44919, Republic of Korea; skiende74@gmail.com; 2 Department of Orthopedic Surgery, Busan Medical Center, Busan 47527, Republic of Korea; drkimminwoo@naver.com; 3 Department of Orthopaedic Surgery, Aerospace Medical Center, Republic of Korea Air Force, Cheongju 28330, Republic of Korea; 4 Department of Orthopaedic Surgery, Seoul National University Hospital, Seoul 03080, Republic of Korea

**Keywords:** scoping review, post-traumatic ankle osteoarthritis, osteochondral lesion of the talus, mesenchymal stromal cells, bone marrow aspirate concentrate, adipose-derived stem cells, intra-articular injection, cartilage repair, PRISMA-ScR

## Abstract

**Background**: Ankle osteoarthritis (OA) differs fundamentally from knee OA: it is predominantly post-traumatic, affects younger and more active patients, and frequently arises from focal osteochondral lesions of the talus (OCLT) rather than diffuse degeneration. Cell-based and orthobiologic therapies—bone marrow aspirate concentrate (BMAC), bone marrow-derived cell transplantation (BMDC), adipose-derived mesenchymal stromal cells (ADMSCs), stromal vascular fraction (SVF), micro-fragmented adipose tissue (mFAT), and peripheral blood-derived products—have been proposed as joint-preserving options, but the evidence base has not been mapped in a way that separates degenerative post-traumatic ankle OA from focal chondral repair, or that distinguishes cells given as a standalone injection from cells given alongside a therapeutic operation. **Methods**: We conducted a systematic scoping review following the PRISMA-ScR framework. Human clinical studies applying cell-based therapies to post-traumatic ankle OA and/or OCLT were charted by population, cell source and preparation, mode of delivery, concomitant procedures, follow-up, and reported clinical and structural outcomes. Mode of delivery—standalone intra-articular injection versus adjunct to a concomitant surgical procedure—was pre-specified as a primary analytic axis, because it determines whether an observed effect can be attributed to the cell product at all. Given anticipated clinical and product heterogeneity and the near-absence of controlled trials of standalone injection, no meta-analysis was undertaken; the objective was to map the evidence, characterise its structure, and identify gaps. **Results**: Eleven clinical studies were charted. The dominant structural feature of this literature is that in nine of 11 studies the cells were co-administered with a therapeutic surgical procedure—marrow stimulation, autologous osteochondral transplantation, supramalleolar or calcaneal osteotomy, or joint debridement—so that in no charted study can the contribution of the cells be separated from that of the operation. This attribution problem, rather than any efficacy estimate, is the principal finding of the review. The remaining evidence is small, heterogeneous, and uniformly non-randomised (Level of Evidence III–V). Only two studies used standalone injection, and for degenerative post-traumatic ankle OA specifically, the standalone-injection evidence consists of a single case report. Reported clinical outcomes (AOFAS, VAS, FAOS/KOOS, Tegner) and structural surrogates (MOCART, T2-mapping, second-look arthroscopy) were generally favourable, and adverse events were mild and self-limiting; however, no study demonstrated histologically confirmed hyaline regeneration, and no adequately powered randomised trial of standalone injection for degenerative post-traumatic ankle OA was identified. **Conclusions**: Cell-based therapies for post-traumatic ankle OA and OCLT show promising but preliminary, hypothesis-generating signals within a fragmented evidence base in which the cellular contribution is confounded by concomitant surgery in nine of 11 charted studies; on present evidence, the field cannot claim an independent effect for the cell product. The knee OA evidence cannot be extrapolated to the ankle because of the joint’s distinct biology, aetiology, and lesion pattern. Adequately powered randomised trials of standalone intra-articular injection in well-defined degenerative post-traumatic ankle OA, using standardised product characterisation and a core outcome set with quantitative structural endpoints, are the principal advance needed.

## 1. Introduction

Osteoarthritis is among the most prevalent musculoskeletal disorders worldwide and a leading cause of chronic pain and disability, with a burden projected to rise markedly through 2050 [1,2]. Within this broad disease category, ankle (tibiotalar) osteoarthritis (OA) occupies a distinctive position. In contrast to the knee and hip, where disease is most often idiopathic, the majority of symptomatic ankle OA is post-traumatic: series from tertiary referral centres attribute roughly three-quarters of end-stage ankle OA to prior trauma, most commonly malleolar and pilon fractures and recurrent ligamentous instability [3,4,5,6]. As a consequence, patients presenting with post-traumatic ankle OA are typically younger and more physically active than those with primary knee OA, and the molecular and tissue-level cascade that follows joint injury differs from that of age-related degeneration [5,7].

The tibiotalar joint is also biologically distinct at the level of the cartilage itself. Compared with knee cartilage, ankle cartilage has a higher proteoglycan and water content, greater matrix stiffness, a higher rate of matrix synthesis, and chondrocytes that are comparatively resistant to catabolic cytokines such as interleukin-1; these properties are thought to underlie the lower baseline incidence of primary OA in the ankle and a potentially greater intrinsic capacity for repair [8,9]. This biology has an important implication for evidence synthesis: findings from the far larger knee OA literature cannot be assumed to transfer to the ankle, and the two joints warrant separate appraisal.

Current management of established ankle OA is limited. Non-operative measures are symptomatic, and no pharmacological agent is approved as a disease-modifying OA drug on the basis of structural cartilage outcomes [10,11]. For end-stage disease, the definitive surgical options remain ankle arthrodesis and total ankle arthroplasty; both relieve pain but carry trade-offs—loss of motion or long-term revision risk—that are especially consequential in the young, active, post-traumatic population that predominates in ankle OA [12]. This therapeutic gap has driven interest in joint-preserving, biologic strategies, among which cell-based and orthobiologic therapies have attracted growing clinical attention.

Mesenchymal stromal cells (MSCs) and related orthobiologic preparations are thought to act predominantly through paracrine and immunomodulatory signalling—suppression of synovial inflammation, chondrocyte protection, and trophic support of endogenous repair—rather than through durable engraftment and direct differentiation [13,14]. Several distinct products have been applied to the ankle: culture-expanded ADMSCs, minimally manipulated adipose preparations (SVF and mFAT), bone marrow aspirate concentrate (BMAC) and one-step bone marrow-derived cell transplantation (BMDC), and, in related joints, peripheral blood-derived products. These differ in progenitor content, mechanism, manufacturing, and regulatory status, and—crucially for the ankle—they have been deployed both as standalone intra-articular injections and as biologic adjuncts to surgical cartilage-repair or realignment procedures.

Prior syntheses have begun to describe this field but have not resolved the central interpretive problem. A previous systematic review of adipose-derived cells for ankle pathology identified only a small number of studies, all of which combined cell therapy with a concomitant procedure, and concluded that clinical benefit could not be attributed to the cells alone [15]. A separate review examined concentrated bone marrow aspirate for OCLT, organised by surgical approach, and reached a comparable conclusion within that single cell source. Each of these is therefore bounded by one cell source; neither maps the field across sources, and neither treats degenerative post-traumatic ankle OA and focal osteochondral repair as separate target conditions. What is missing is a synthesis that maps the whole cell-based evidence base for the ankle, explicitly separates degenerative post-traumatic ankle OA from focal osteochondral repair, and distinguishes standalone injection from surgical-adjunct use. Because the anticipated heterogeneity and scarcity of controlled data make a valid meta-analysis inappropriate, a scoping review is the methodologically appropriate approach: its purpose is to map the nature, extent, and structure of the evidence and to define the questions that future trials must answer [16,17]. Accordingly, the objective of this study is to chart the clinical evidence for cell-based therapies in post-traumatic ankle OA and OCLT, to characterise how that evidence is structured, and to identify the gaps that currently preclude comparative, mechanism-anchored conclusions.

## 2. Materials and Methods

### 2.1. Protocol and Reporting

This review was designed and reported in accordance with the PRISMA Extension for Scoping Reviews (PRISMA-ScR) [16] and the methodological framework of Arksey and O’Malley as refined for scoping reviews [17]; the PRISMA 2020 statement informed the flow reporting where applicable [18]. A review protocol defining the objective, eligibility criteria, and charting approach was prepared a priori. Scoping reviews are eligible for registration in PROSPERO [CRD420251184655]; the protocol is available from the corresponding author on request.

### 2.2. Eligibility Criteria (Population–Concept–Context)

Population: adults (≥18 years) with post-traumatic ankle (tibiotalar) OA of any radiographic grade and/or osteochondral lesions of the talus (OCLT), including lesions of post-traumatic origin. Concept: any cell-based or cell-containing orthobiologic therapy delivered to the ankle—culture-expanded ADMSCs or BMMSCs, SVF, mFAT, BMAC, one-step BMDC, or peripheral blood-derived products—whether administered as a standalone intra-articular injection or as an adjunct to a surgical procedure. Context: human clinical studies (randomised controlled trials, prospective and retrospective comparative studies, prospective single-arm series, and case reports/series) reporting clinical and/or structural outcomes. Pre-clinical (animal or in vitro) studies, narrative reviews, and conference abstracts without extractable data were excluded from charting but were used to inform the background and discussion. Each study was tagged by indication (degenerative post-traumatic ankle OA vs. focal OCLT) and by mode of delivery (standalone injection vs. surgical adjunct).

### 2.3. Information Sources and Search Strategy

**Figure 1:**
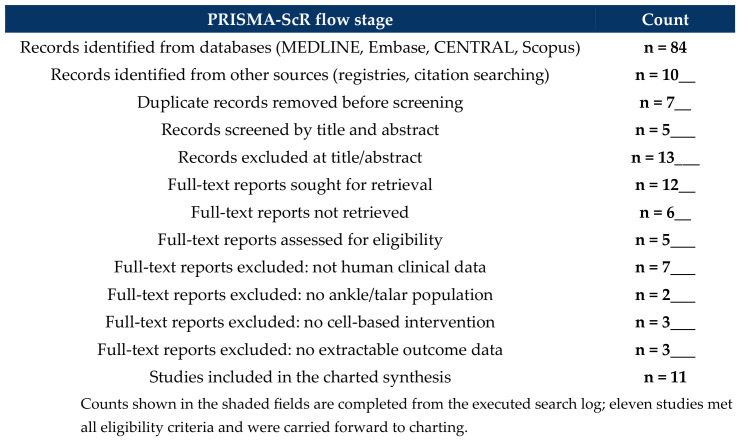
PRISMA-ScR flow of study identification, screening, and inclusion.

Electronic searches of MEDLINE (via PubMed), Embase, Cochrane CENTRAL, and Scopus were executed from database inception to 01 March 2026, without language restriction. The strategy combined three conceptual blocks: (1) cell-source terms (adipose-derived stem cell OR stromal vascular fraction OR micro-fragmented adipose tissue OR bone marrow mesenchymal stem cell OR bone marrow aspirate concentrate OR bone marrow-derived cell OR peripheral blood stem cell); (2) anatomical and disease terms (ankle OR tibiotalar OR talus OR talar OR osteochondral lesion); and (3) intervention terms (injection OR transplantation OR implantation). Blocks were combined with AND and terms within blocks with OR, using both controlled vocabulary (MeSH/Emtree) and free-text fields; the complete, database-specific search strings are provided as Supplementary Table S1. ClinicalTrials.gov and the reference lists of eligible studies and relevant reviews were screened manually. Records were deduplicated in a reference manager before screening. The resulting study-selection process is reported in full in the PRISMA-ScR flow diagram (Figure 1).

### 2.4. Study Selection and Data Charting

Titles/abstracts and then full texts were screened independently by two reviewers (S.Y.J. and D.H.L.) against the eligibility criteria, with disagreements resolved by a third reviewer (M.W.K.). A standardised charting form captured study design and level of evidence; participant number and characteristics; cell source, preparation, and dose; mode of delivery and any concomitant procedure; indication (degenerative post-traumatic ankle OA vs. OCLT); comparator; follow-up; and clinical and structural outcomes.

### 2.5. Critical Appraisal

Consistent with scoping-review methodology, formal critical appraisal is optional; we nonetheless summarised methodological limitations to contextualise the evidence. Non-randomised studies were appraised using the Methodological Index for Non-Randomised Studies (MINORS) [19] and, where applicable, ROBINS-I [20]; any randomised trials would be appraised with RoB 2 [21]. Appraisal was used descriptively to characterise the evidence, not to weight or pool it.

### 2.6. Synthesis

A descriptive, narrative synthesis structured by cell source, indication, and mode of delivery was undertaken. A quantitative meta-analysis was deliberately not performed: the studies are few, largely non-randomised, and dominated by cell delivery as a surgical adjunct, so pooling would violate the assumptions of a valid meta-analysis and would misattribute the effect of concomitant procedures to the cell product. Formal certainty-of-evidence grading (e.g., GRADE [22]) was likewise not applied, consistent with scoping-review methodology, whose aim is to map rather than to grade a pooled estimate. This constraint is itself a principal finding of the review.

## 3. Results

### 3.1. Structure of the Evidence Base and Attribution of Effect

Eleven clinical studies met the eligibility criteria and were charted (Table 1; full study-level detail in Supplementary Table S1). One finding dominates and governs the interpretation of everything that follows: in nine of the 11 studies, the cell product was co-administered with a therapeutic surgical procedure—arthroscopic marrow stimulation, autologous osteochondral transplantation, one-step scaffold implantation, joint debridement, or realignment osteotomy. In none of these studies was a design element present that could separate the contribution of the cells from that of the operation. Only two studies delivered cells as a standalone injection, and of these only one addressed degenerative post-traumatic ankle OA—a single case report. The evidence base therefore does not currently permit any statement about the independent efficacy of cell therapy in the ankle, and this attribution problem is reported here as the principal result rather than as a limitation. Two further features characterise the evidence. It is methodologically limited, consisting of retrospective comparative studies, prospective single-arm series, and case reports (Level of Evidence III–V), with no adequately powered randomised controlled trial identified. It is also heterogeneous in target condition, spanning focal OCLT in younger patients (often of post-traumatic aetiology) and diffuse degenerative post-traumatic ankle OA; these differ in pathophysiology and treatment objective and are charted separately here rather than conflated.

### 3.2. Bone Marrow-Derived Therapies (BMDC and BMAC)

The most developed body of ankle evidence concerns one-step bone marrow-derived cell transplantation (BMDC) for focal OCLT. In prospective single-arm series, arthroscopic BMDC combined with a biomaterial scaffold produced clinically meaningful AOFAS improvement, with hyaline-like repair tissue documented on MRI and, in a subset, histology [23,24,25]. A characteristic temporal pattern was observed: outcomes peaked at around 24 months and then declined gradually over longer follow-up, with lesion area the dominant predictor of the final score [24]. When the same technique was applied to OCLT occurring in the setting of concomitant ankle OA, combined with joint debridement, mean AOFAS improved to 24 months and then trended downward, reaching 77.8 ± 18.3 at 36 months—consistent with symptomatic benefit that attenuates as background degeneration progresses [26]. As an augment to autologous osteochondral transplantation, concentrated bone marrow aspirate (CBMA) was associated with a lower rate of postoperative subchondral cyst formation at a minimum of five years, although functional scores did not differ significantly from transplantation alone [27]; a recent systematic review of CBMA for OCLT similarly concluded that its value lies chiefly in an adjunctive role rather than as a standalone therapy. In the distinct entity of early, pre-collapse post-traumatic talar osteonecrosis, percutaneous autologous bone marrow concentrate reduced progression to collapse and arthrodesis relative to core decompression alone [28]—one of only two charted studies in which cells were given without a concomitant operation.

### 3.3. Adipose-Derived Therapies (SVF, mFAT, ADMSC)

Adipose-derived preparations have been applied to the ankle chiefly as an adjunct to marrow stimulation for OCLT. In a comparative cohort, addition of a stromal vascular fraction containing ADMSCs to arthroscopic marrow stimulation improved pain and function beyond marrow stimulation alone (VAS 7.1→3.9 vs. 7.1→4.5; AOFAS 68.5→78.3 vs. 67.7→76.9) and yielded superior MOCART cartilage-repair scores, with the benefit most apparent for larger lesions [29]. Standalone intra-articular adipose therapy for degenerative post-traumatic ankle OA was represented only at the case level: a single patient with end-stage post-traumatic ankle OA treated with autologous mFAT (Lipogems) reported improved VAS, MOXFQ, and FAAM at six months without complication, framed as a potential means of delaying fusion or replacement in a young patient [31]. A prior systematic review of adipose-derived cells for ankle pathology reached the same structural conclusion reached here—that all included studies combined cell therapy with a concomitant procedure, precluding attribution of benefit to the cells alone [15].

### 3.4. Cell Therapy as an Adjunct in Realignment Surgery for Varus Ankle OA

A further cluster of studies used cell injection to augment realignment surgery for varus ankle OA. Adding ADMSC or MSC injection to arthroscopic marrow stimulation performed alongside lateral sliding calcaneal osteotomy [30] or supramalleolar osteotomy [33] improved second-look arthroscopic cartilage regeneration and clinical outcomes relative to marrow stimulation without cells. MSC injection with arthroscopic treatment was also effective in older patients with OCLT, indicating that advanced age is not by itself a contraindication [32]. However, in every case, the cells were one component of a multi-procedure intervention, so their independent contribution remains unquantified.

### 3.5. Structural and Imaging Outcomes

Structural assessment across the mapped studies relied on MOCART scoring, quantitative T2-mapping, and second-look arthroscopy, several of which favoured cell-augmented over control treatment and, in the BMDC series, showed hyaline-like repair with T2-mapping capable of predicting outcome [25,29,30]. However, no study demonstrated complete histological restoration of hyaline cartilage. Structural findings are therefore best interpreted at the level of imaging-based and second-look structural modification rather than confirmed hyaline regeneration.

### 3.6. Safety

Reported adverse events were mild and self-limiting across sources, comprising injection-site or harvest-site discomfort, transient effusion, and swelling; no malignant transformation or serious treatment-related event was reported. Procedures involving bone marrow or adipose harvest carry the additional, generally minor, morbidity of the donor site. This favourable short- to mid-term safety profile is consistent with the broader intra-articular orthobiologic literature, although heterogeneous reporting warrants continued vigilance.

## 4. Discussion

### 4.1. Summary of Findings

This scoping review maps the clinical evidence for cell-based therapies in post-traumatic ankle OA and OCLT and reaches four principal conclusions. First, and most importantly, the defining structural feature of this literature is that in nine of 11 charted studies cells were delivered alongside a therapeutic operation rather than as a standalone injection, so the independent effect of the cell product cannot be isolated in any of them. Second, the evidence base is small, heterogeneous, and methodologically limited, without adequately powered randomised trials. Third, the target condition is not uniform: focal OCLT in young, often post-traumatic patients has been studied far more than diffuse degenerative post-traumatic ankle OA, for which only case-level evidence of standalone injection exists. Fourth, within these constraints, the reported clinical and imaging signals are consistently favourable, and the safety profile is benign, which justifies—but does not substitute for—dedicated trials.

### 4.2. Relationship to Previous Syntheses and to Adjacent Fields

It is worth stating precisely how this review differs from earlier reports in allied domains. Arceri et al. restricted their systematic review to adipose-derived cells applied to ankle pathology, identified eight studies, and found that all combined cell therapy with a concomitant procedure [15]. Wagner et al. examined concentrated bone marrow aspirate for OCLT, organised by surgical approach, and likewise concluded that its demonstrated value is adjunctive. Each is bounded by a single cell source. Syntheses of intra-articular cell therapy in the knee [14,34,35,36,37,38] address a different joint, with a different aetiology, cartilage biology, and lesion pattern. The present review differs in three specific respects: it maps the ankle evidence across all cell sources simultaneously rather than one at a time; it uses mode of delivery (standalone injection versus surgical adjunct) and indication (degenerative OA versus focal OCLT) as explicit, pre-specified charting axes; and it therefore renders the attribution problem quantifiable—nine of 11 studies—rather than leaving it as a narrative caveat at the end of a discussion.

The wider regenerative-medicine literature is relevant here as context rather than as evidence. Work on chronic wound repair has shown that the delivery vehicle itself can be engineered to modulate the local oxidative and immune microenvironment—for instance, hydrogel systems designed to neutralise reactive oxygen species and to shift macrophage polarisation toward a reparative phenotype [39,40]. These are wound-healing platforms, and their clinical findings are not transferable to the tibiotalar joint. However, they do illustrate a principle that bears directly on the scaffold-supported one-step BMDC techniques charted here: the carrier is not biologically inert, and the immunomodulatory properties of the scaffold may contribute to the observed effect independently of the cells it delivers. This constitutes a further and, to date, unexamined layer of the attribution problem in the ankle literature, and it argues for trial designs that include a cell-free scaffold arm.

### 4.3. Why the Knee Evidence Cannot Be Extrapolated to the Ankle

The temptation to transfer the comparatively mature knee OA evidence [14,34,35,36], including an expanding knee bone marrow aspirate concentrate literature [37,38], to the ankle should be resisted. The joints differ in aetiology (predominantly post-traumatic and younger in the ankle [3,4,5,6]), in cartilage biology (higher proteoglycan content, greater matrix stiffness, and cytokine-resistant chondrocytes in the ankle [8,9]), and in the dominant lesion pattern (focal osteochondral defects rather than diffuse degeneration). These differences plausibly modify both the natural history and the response to biologic therapy, and they explain why an intervention with moderate-certainty support in the knee cannot be assumed effective in the ankle. Separate appraisal, as undertaken here, is therefore essential.

### 4.4. The Surgical-Adjunct Confound

The most consequential interpretive limitation of the ankle literature is that cell therapy is typically bundled with a procedure that is itself therapeutic—marrow stimulation, osteochondral transplantation, scaffold implantation, or realignment osteotomy [23,24,25,26,27,29,30,32,33]. Marrow stimulation in particular recruits endogenous marrow-derived cells to the defect, so a co-administered cell product and the host response act on the same target, and observed improvements cannot be partitioned between them. This is not a peripheral caveat: it is the reason a meta-analysis is inappropriate and the reason that, despite uniformly positive reports, the field cannot yet claim an independent effect for the injected cells. A previous adipose-cell review reached the identical conclusion [15], and our broader mapping shows the problem to be general across cell sources. This confound is compounded by the fact that the comparators against which cell therapy is judged are themselves imperfectly characterised: bone marrow stimulation and microfracture for OCLT show durable but gradually attenuating outcomes over long-term follow-up [41,42], and cell-free polymer scaffolds provide a further active benchmark [43], so a positive result relative to any of these does not establish an independent cellular effect.

### 4.5. Product and Indication Heterogeneity

The products grouped under “cell therapy” differ substantially. Culture-expanded MSCs deliver a defined dose but require manufacturing infrastructure and advanced-therapy regulation; minimally manipulated preparations (SVF, mFAT, BMAC, one-step BMDC) can be produced at the point of care but deliver a variable, undefined cell content [13]. Layered onto this is indication heterogeneity: focal OCLT and diffuse degenerative ankle OA differ in pathophysiology and objective. Pooling across products and indications would obscure rather than clarify, and the current evidence is too sparse to support the subgroup analyses that would be needed to resolve these distinctions.

### 4.6. Mechanistic Considerations

Across sources, the dominant mechanism is now understood to be paracrine and immunomodulatory—trophic support of endogenous repair and suppression of the inflammatory milieu—rather than durable engraftment and direct chondrogenesis [13,44,45]. This is consistent with the pattern seen in the ankle data: symptomatic and imaging-based improvement without confirmed hyaline regeneration, and, in the BMDC series, an early peak followed by gradual decline as background degeneration proceeds [24,26]. In the setting of marrow stimulation, exogenous cells may act principally by augmenting a repair response that the procedure has already initiated, which further entangles their effect with that of the surgery.

### 4.7. Gaps and Future Directions

Several concrete steps would move the field forward. First, adequately powered randomised trials of standalone intra-articular injection in clearly defined degenerative post-traumatic ankle OA are needed, with an inert or standard-of-care comparator, so that the cellular effect can be isolated from concomitant surgery. Where cells are given with a scaffold, a cell-free scaffold arm should be included so that carrier and cell effects can be separated. Second, focal OCLT and degenerative ankle OA should be enrolled and analysed separately, with pre-specified lesion characterisation. Third, products should be characterised prospectively using mechanism-aligned potency assays consistent with the 2025 ISCT framework [13], with sufficient manufacturing detail to separate culture-expanded from minimally manipulated preparations. Fourth, trials should adopt a validated ankle-specific core outcome set (for example FAOS or AOFAS as the primary clinical endpoint) alongside quantitative structural endpoints (T2 or T1-rho mapping, MOCART) so that structural modification can be distinguished from symptomatic benefit. Fifth, a minimum follow-up of 24 months and prospective registries are required to establish durability and long-term safety, particularly given the young age of this population and the observed late attenuation of benefit.

### 4.8. Limitations

This review has limitations. As a scoping review, it maps and characterises the evidence but does not grade a pooled effect; by design it makes no quantitative efficacy claim. The included evidence is dominated by non-randomised, surgical-adjunct studies, and the near-absence of standalone-injection trials for degenerative post-traumatic ankle OA limits any inference about injection therapy specifically. Small or non-English studies may have been under-captured despite the absence of language restriction, and publication bias—positive single-arm series being more likely to appear—cannot be excluded and may inflate the apparent consistency of benefit.

## 5. Conclusions

Cell-based therapies for post-traumatic ankle osteoarthritis and osteochondral lesions of the talus are supported by promising but preliminary and hypothesis-generating evidence. The literature is small, heterogeneous, and structurally confounded, because cells are in 9 of 11 charted studies delivered as an adjunct to a therapeutic surgical procedure rather than as a standalone injection, and because focal osteochondral repair and diffuse degenerative disease are frequently conflated. Reported clinical and imaging outcomes are favourable and the safety profile is benign, but no study has demonstrated confirmed hyaline regeneration, and the independent contribution of the cell product remains unquantified. Because the ankle differs from the knee in aetiology, cartilage biology, and lesion pattern, the more mature knee evidence cannot be transferred. Adequately powered randomised trials of standalone intra-articular injection in well-defined degenerative post-traumatic ankle OA, with standardised product characterisation, an ankle-specific core outcome set, quantitative structural endpoints, and follow-up of at least 24 months, are the decisive advance the field now requires.

## Figures and Tables

**Table 1:** Summary of charted clinical studies, grouped by cell source. Studies are ordered within each group; bracketed numbers correspond to the reference list. Where two counts are given (e.g., 28 vs. 26), they denote the cell-treated and comparator arms. Full study-level detail—including preparation method, individual concomitant procedures, and follow-up duration—is provided in Supplementary Table S1.

Study (Year)	Design (LoE)	*n*	Delivery Mode and Concomitant Procedure	Indication	Key Reported Outcome
A. Bone marrow-derived preparations (one-step BMDC, BMAC/CBMA)					
Giannini et al. (2009) [23]	Prospective case series (IV)	48	Surgical adjunct: arthroscopic one-step BMDC + scaffold	Focal talar OCL	AOFAS improved; hyaline-like repair on MRI/histology in a subset
Buda et al. (2013) [24]	Prospective case series (IV)	64	Surgical adjunct: one-step BMDC + scaffold	Focal talar OCL	Peak at 24 mo, decline to AOFAS ~80 at 72 mo; lesion area main predictor
Giannini et al. (2013) [25]	Prospective case series (IV)	Talar OCL cohort	Surgical adjunct: one-step BMDC + scaffold	Focal talar OCL	Sustained improvement at 4 yr; T2-mapping predicted outcome
Buda et al. (2016) [26]	Prospective case series (IV)	56	Surgical adjunct: one-step BMDC + joint debridement	Talar OCL with concomitant ankle OA	AOFAS 77.8 ± 18.3 at 36 mo; declining trend after 24 mo
Shimozono et al. (2019) [27]	Retrospective comparative (III)	28 vs. 26	Surgical adjunct: CBMA + autologous osteochondral transplantation	Focal talar OCL	Fewer postoperative subchondral cysts with CBMA; FAOS/SF-12 similar
Hernigou et al. (2018) [28]	Comparative cohort (III)	45 vs. 34	Standalone: percutaneous BMAC vs. core decompression	Early pre-collapse post-traumatic talar osteonecrosis	Fewer progressions to collapse/arthrodesis than core decompression alone
B. Adipose-derived preparations (SVF, ADMSC, mFAT)					
Kim et al. (2014) [29]	Cohort (III)	24 vs. 26 ankles	Surgical adjunct: SVF injection + arthroscopic marrow stimulation	Focal talar OCL	VAS 7.1→3.9 vs. 7.1→4.5; AOFAS 68.5→78.3 vs. 67.7→76.9; better MOCART
Kim & Koh (2016) [30]	Retrospective comparative (III)	26 vs. 23	Surgical adjunct: ADMSC injection + calcaneal osteotomy + marrow stimulation	Varus ankle OA	Superior second-look cartilage regeneration; improved VAS/AOFAS
Niazi et al. (2021) [31]	Case report (V)	1	Standalone: intra-articular mFAT (Lipogems)	End-stage post-traumatic ankle OA	Improved VAS, MOXFQ, FAAM at 6 mo; no complications
C. Mesenchymal stromal cell injection (preparation as reported in source)					
Kim et al. (2013) [32]	Retrospective comparative (III)	Older-patient OCLT cohort	Surgical adjunct: MSC injection + arthroscopic treatment	Focal talar OCL (older patients)	Improved AOFAS/VAS; age not a contraindication
Kim, Lee & Koh (2016) [33]	Retrospective comparative (III)	31 vs. 33 ankles	Surgical adjunct: MSC injection + supramalleolar osteotomy + marrow stimulation	Varus ankle OA	Improved cartilage regeneration and clinical outcomes

AOFAS, American Orthopaedic Foot and Ankle Society score; ADMSC, adipose-derived mesenchymal stromal cell; BMAC, bone marrow aspirate concentrate; BMDC, bone marrow-derived cell; CBMA, concentrated bone marrow aspirate; FAAM, Foot and Ankle Ability Measure; FAOS, Foot and Ankle Outcome Score; LoE, level of evidence; mFAT, micro-fragmented adipose tissue; MOCART, Magnetic Resonance Observation of Cartilage Repair Tissue; MOXFQ, Manchester–Oxford Foot Questionnaire; OA, osteoarthritis; OCL (T), osteochondral lesion (of the talus); SVF, stromal vascular fraction; VAS, visual analogue scale.

## Data Availability

No new data were created or analysed in this study. All data derive from the individual studies cited in the reference list.

## Supplementary Materials

The following supporting information can be downloaded at: https://www.mdpi.com/article/10.3390/bioengineering13070843/s1. Supplementary Table S1 (full study-level charting).
