# Supplementary material for: Cell-Based Therapies for Post-Traumatic Ankle Osteoarthritis and Osteochondral Lesions of the Talus: A Systematic Scoping Review of an Emerging and Heterogeneous Evidence Base
Source: Bioengineering (Basel). 2026 Jul 22;13(7):843. doi: 10.3390/bioengineering13070843 (PMC13403794; doi:10.3390/bioengineering13070843)
Supplement: Supplementary file 1 [file bioengineering-13-00843-s001.zip › bioengineering-4442457-supplementary.pdf]

Supplementary Table S1.

Full study-level charting of clinical studies of cell-based therapies for post-traumatic ankle osteoarthritis and osteochondral lesions of the talus. This table carries the expanded detail previously contained in the main-text table; the condensed, grouped summary appears as Table 1.

Study (Year)	Design (LoE)	n (cell/total)	Cell source/preparation	Concomitant procedure	Indication	Follow-up	Key reported outcome
Giannini et al. (2009) [23]	Prospective case series (IV)	48/48	One-step arthroscopic autologous BMDC + biomaterial scaffold	Arthroscopic implantation/marrow-derived cell scaffold	Focal talar OCL (chronic type II)	24–36 mo	AOFAS improvement; hyaline-like repair by MRI/histology in a subset
Buda et al. (2013) [24]	Prospective case series (IV)	64/64	One-step BMDC + scaffold (two scaffolds)	Arthroscopic scaffold implantation	Focal talar OCL	Mean 53 mo	AOFAS peaked at 24 mo, gradual decline to ~80 at 72 mo; lesion area was main predictor
Giannini et al. (2013) [25]	Prospective case series (IV)	Talar OCL cohort	One-step BMDC + scaffold	Arthroscopic scaffold implantation	Focal talar OCL	4 yr	Sustained clinical improvement; T2-mapping predicted outcome
Buda et al. (2016) [26]	Prospective case series (IV)	56/56 (mean age 35.6)	One-step BMDC transplantation	Joint debridement	Talar OCL with concomitant ankle OA	36 mo	AOFAS 77.8 ± 18.3 at 36 mo (improvement to 24 mo, declining trend thereafter)
Shimozono et al. (2019) [27]	Retrospective comparative (III)	28 vs 26/54	Concentrated bone marrow aspirate (CBMA) augmenting AOT	Autologous osteochondral transplantation	Focal talar OCL	Min 5 yr	Lower postoperative subchondral cyst occurrence with CBMA; FAOS/SF-12 similar
Kim et al. (2013) [32]	Retrospective comparative (III)	Older-patient OCLT cohort	MSC injection with arthroscopic treatment	Arthroscopic marrow stimulation	Focal talar OCL (older patients)	Mid-term	Improved AOFAS/VAS; age not a contraindication to MSC augmentation
Kim et al. (2014) [29]	Cohort (III)	24 vs 26/50 ankles	SVF containing ADMSCs, injected	Arthroscopic marrow stimulation	Focal talar OCL	Mean ~2 yr	VAS 7.1→3.9 vs 7.1→4.5; AOFAS 68.5→78.3 vs 67.7→76.9; better MOCART with SVF
Kim & Koh (2016) [30]	Retrospective comparative (III)	26 vs 23/49	ADMSC injection (marrow-stimulation adjunct)	Lateral sliding calcaneal osteotomy + marrow stimulation	Varus ankle OA	Second-look ~12 mo	MSC group superior second-look cartilage regeneration; improved VAS/AOFAS
Kim, Lee & Koh (2016) [33]	Retrospective comparative (III)	31 vs 33/64 ankles	MSC injection (marrow-stimulation adjunct)	Supramalleolar osteotomy + marrow stimulation	Varus ankle OA	Mean 12.8 mo	Additional MSC injection improved cartilage regeneration and clinical outcomes
Niazi et al. (2021) [31]	Case report (V)	1/1 (age 39)	Autologous micro-fragmented adipose tissue (Lipogems)	None (standalone IA injection)	End-stage post-traumatic ankle OA	6 mo	Improved VAS, MOXFQ and FAAM; no complications; potential to delay fusion/replacement
Hernigou et al. (2018) [28]	Comparative cohort (III)	45 vs 34	Percutaneous autologous bone marrow concentrate (~124×10³ MSC/ankle)	None (percutaneous injection) vs core decompression control	Early post-traumatic talar osteonecrosis (pre-collapse)	Long-term	Fewer progressions to collapse/arthrodesis vs core decompression alone

Abbreviations as in Table 1. AOT, autologous osteochondral transplantation; IA, intra-articular; mo, months; yr, years.
